# Enhanced Vapor Transmission Barrier Properties via Silicon-Incorporated Diamond-Like Carbon Coating

**DOI:** 10.3390/polym13203543

**Published:** 2021-10-14

**Authors:** Parand R. Riley, Pratik Joshi, Sina Azizi Machekposhti, Ritesh Sachan, Jagdish Narayan, Roger J. Narayan

**Affiliations:** 1 Department of Materials Science and Engineering, Centennial Campus, North Carolina State University, Raleigh, NC 27695-7907, USA; prostam@ncsu.edu (P.R.R.); pjoshi5@ncsu.edu (P.J.); narayan@ncsu.edu (J.N.); 2 Joint Department of Biomedical Engineering, Centennial Campus, North Carolina State University, Raleigh, NC 27695-7115, USA; sazizim@ncsu.edu; 3 Department of Mechanical Engineering, Oklahoma State University, Stillwater, OK 74078, USA; rsachan@okstate.edu

**Keywords:** coating, surface structure, moisture vapor transmission, silicon-incorporated diamond-like carbon, plasma-enhanced chemical vapor deposition, water stability

## Abstract

In this study, we describe reducing the moisture vapor transmission through a commercial polymer bag material using a silicon-incorporated diamond-like carbon (Si-DLC) coating that was deposited using plasma-enhanced chemical vapor deposition. The structure of the Si-DLC coating was analyzed using scanning electron microscopy, Raman spectroscopy, X-ray photoelectron spectroscopy, energy-dispersive X-ray spectroscopy, selective area electron diffraction, and electron energy loss spectroscopy. Moisture vapor transmission rate (MVTR) testing was used to understand the moisture transmission barrier properties of Si-DLC-coated polymer bag material; the MVTR values decreased from 10.10 g/m^2^ 24 h for the as-received polymer bag material to 6.31 g/m^2^ 24 h for the Si-DLC-coated polymer bag material. Water stability tests were conducted to understand the resistance of the Si-DLC coatings toward moisture; the results confirmed the stability of Si-DLC coatings in contact with water up to 100 °C for 4 h. A peel-off adhesion test using scotch tape indicated that the good adhesion of the Si-DLC film to the substrate was preserved in contact with water up to 100 °C for 4 h.

## 1. Introduction

Diamond-like carbon (DLC) is an amorphous variety of carbon made up of trigonally and tetrahedrally hybridized carbon atoms; nanoscale or microcrystalline graphitic regions are commonly noted within the amorphous matrix [1]. In 1971, Aisenberg et al. grew amorphous and insulating carbon coatings using a beam of carbon ions that was created in an argon plasma; the term “diamond-like” was used to indicate that the properties of this coating are similar to those of diamond [2]. The deposition of DLC coatings requires an energy source that is used to generate excited carbon species from laser-carbon target interaction, arc discharge, acceleration of carbon ions, or interaction with energetic ions (e.g., sputtering). 

DLC has several positive attributes as a barrier coating: (a) it exhibits a high atomic density, (b) it can be deposited on a temperature-sensitive (e.g., polymer) surface, (c) it can be deposited at low cost using cost-effective precursor materials [3,4,5], and (d) DLC is a promising material that is being considered for biocompatible barrier applications alongside carbon nanostructures such as graphene and fullerenes [6]. Several studies have considered the use of DLC as a barrier coating [3,4,5]. In 2004, Abbas et al. described the use of hydrogenated DLC coatings deposited by radio frequency plasma-enhanced chemical vapor deposition to improve the gas barrier properties of polycarbonate and polyethylene terephthalate [4]. The lower density hydrogenated DLC coating was shown to impart better gas barrier activity, as evidenced by a lower water vapor transmission rate, than the higher density hydrogen-free DLC coating.

In another study, Boutroy et al. examined the gas barrier properties of hydrogenated DLC coatings that were grown on polyethylene terephthalate substrates using a microwave plasma-enhanced chemical vapor deposition process that was optimized for coating bottles in a short amount of time [5]. Using a Mocon gas permeation instrument, they showed that a 40 nm thick hydrogenated DLC coating significantly reduced oxygen transmission. They demonstrated that an increase in shelf life of beer and soft drinks could be obtained through the use of the hydrogenated DLC coating. Casiraghi et al. subsequently showed that microwave plasma-enhanced chemical vapor deposition is able to (a) coat bottles at a relatively high rate of 60 nm/s and (b) uniformly coat bottles, with only a 15% variation in thickness over the length of the bottle [3]. Zhang et al. coated polyethylene terephthalate with hydrogenated DLC using radio frequency plasma-enhanced chemical vapor deposition [3]; they used a radio frequency (RF) power of 600 W, an argon:acetylene ratio of 1:2, and a gas flow rate of 40 SCCM to create hydrogenated DLC coatings and demonstrated the barrier properties of the hydrogenated DLC coatings.

Silicon-incorporated DLC coatings represent another potentially suitable material for use in barrier applications. In a recent study, the structure and cell viability properties of Si-DLC coatings on fused silica substrates were demonstrated [7]. X-ray photoelectron spectroscopy (XPS) indicated that the coatings contained carbon, oxygen, and silicon; sp^2^ (C=C), sp^3^ (C-C), C-O, and C=O bonds were noted in the coatings. Fourier transform infrared spectroscopy (FTIR) analysis showed the presence of spectral features associated with C-OH stretching, Si-CH_2_ bending, and C-H bending. L929 fibroblast-like cells showed no statistically significant difference in cell viability when cultured on Si-DLC coatings or uncoated fused silica [7].

The water barrier properties of DLC coatings have previously been demonstrated. For example, additively manufactured polymers suffer from high water permeation rates, which limit their functionality and shelf life. Dangnan et al. reported a water vapor transmission rate (WVTR) reduction of up to 70% by applying nitrogen-modified DLC coatings to additively manufactured polymers [8]. The reduction in the water permeation rate was associated with the diffusion barrier properties of the amorphous hydrogenated carbon (a-C:H) coatings [9]. Abbas et al. investigated the water vapor permeation of silicon-doped hydrogenated amorphous carbon (Si-a:C:H) coatings. The Si-a:C:H coatings exhibited a significantly lower WVTR value (0.03 g/m^2^ 24 h) in comparison with the undoped DLC coatings (1.3 g/m^2^ 24 h). This result was attributed to the internal stress reduction in the DLC coating that was associated with the incorporation of silicon [10].

In the present work, silicon-incorporated diamond-like carbon (Si-DLC) coatings were deposited on a commercial polymer material in the shape of a cylindrical bag using plasma-enhanced chemical vapor deposition (PECVD) with a silicon-containing precursor. In addition to materials characterization of the Si-DLC coating, the moisture vapor transmission rate of Si-DLC coated polymer material was compared with that of the uncoated material. These results showed the effectiveness of the Si-DLC coating for reducing the moisture vapor transmission rate of the commercial polymer from 10.10 g/m^2^ 24 h to 6.31 g/m^2^ 24 h. The successful reduction in the MVTR value was associated with stress reduction in the DLC coatings; enhanced adhesion and decreased crack formation in the coating was associated with the incorporation of silicon. The use of the PECVD process with silicon-containing precursors is an effective approach to cover large area surfaces with adherent Si-DLC coatings.

## 2. Materials and Methods

**Figure 1:**
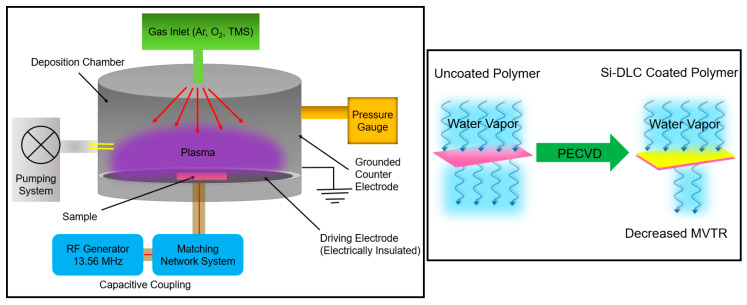
Schematic of the PECVD system used for the deposition of the Si-DLC coatings (left box); the use of the Si-DLC coating to decrease the moisture vapor transmission rate (right box).

The commercial polymer material in a cylindrical shape with a diameter of 20.3 cm and a height of 17.8 cm (ILC Dover, Federica, DE, USA) and Si (100) substrates were cleaned via ultrasonication in an acetone bath for 5 minutes and then in a methanol bath for 5 min; the substrates were immediately transferred to the chamber of the PECVD system for deposition of the Si-DLC coating. A RF plasma-enhanced chemical vapor deposition (PECVD) system was used to deposit Si-DLC coatings on the substrates. The PECVD instrument is an in-house assembled system that is designed for plasma generation in the capacitively coupled mode. The system contains a stainless-steel cylindrical chamber that is customized for the deposition of coatings on large substrates. In the capacitively coupled mode, the driving electrode with a diameter of 12 inches performs as the substrate holder and is located on the bottom plate of the chamber. The electrode is attached to an RFX-600 power supply with a 13.56 MHz frequency, which is electrically insulated from the remainder of the chamber and serves as the counter electrode; the counter electrode is grounded. The chamber is equipped with a water-coolingsystem. The pumping system provides a base pressure of approximately 2 × 10^−8^ Torr. The process gases flowed inside the chamber from above and through a showerhead distribution ring (Figure 1). The substrates were loaded on the chamber base and were in direct contact with the planar electrode. The deposition process was comprised of loading, plasma cleaning, plasma deposition, and unloading steps. After a pump-down for at least three hours that allowed a base pressure of 10^−8^ Torr to be attained, a plasma cleaning step was performed for 10 min using argon and oxygen gases with a mass flow rate of 90 SCCM and 50 SCCM, respectively. During the cleaning step, the peak-to-peak voltage (V_pp_) was kept at 400 ± 10 V, which led to an RF power of 81 ± 10 W and a DC bias of −140 ± 10 V. The Si-DLC coating was deposited using 1.6 SCCM of tetramethylsilane (TMS) and 90 SCCM of argon. The deposition step was performed for 60 minutes with a V_pp_ of 300 ± 10 V, leading to an RF power of 117 ± 10 W and a DC bias of 149 ± 10 V. During cleaning and deposition steps, the total pressure was maintained at 50 mTorr as measured by a Baratron gauge (MKS Instruments, Andover, MA, USA).

The thickness of the Si-DLC coatings was evaluated using the conventional step height approach with a Dektak D150 contact profilometer (Veeco, Plainview, NY, USA) with a tip size radius of 12.5 µm. The thickness of five different points of the coating was measured and averaged. Since the thickness measurements of Si-DLC coating on the polymer material were difficult to perform and the results were inconclusive, thickness measurements were performed on identical Si-DLC coatings that were grown on silicon (100) substrates. The morphology and roughness of the Si-DLC coatings on polymer material and silicon substrates were examined using an MFP-3D Origin+ atomic force microscope (AFM) (Asylum Research, Goleta, CA, USA) in tapping mode with a resonant frequency of approximately 50 kHz, a scan size of 2 µm × 2 µm, and a scan rate of 0.75 lines per second. A Verios 460L field emission scanning electron microscope (FESEM) (FEI, Waltham, MA, USA) with a resolution of 0.6 nm was used to understand the surface morphology of the Si-DLC coatings on the polymer material and silicon substrates. The bonding characteristics of the DLC coating on the silicon substrate were investigated using an alpha300 M Raman microscope (WITec, Ulm, Germany). The Raman system was operated with a solid-state green light laser (=532 nm), a spot size of ~2 µm diameter, and a UHTS 300 spectrometer (WITec, Ulm, Germany). The Raman instrument was calibrated with the 520.6 cm^−1^ peak that is associated with the silicon wafer. X-ray photoelectron spectroscopy (XPS) was performed to understand the carbon bonding hybridization and the elemental composition of the Si-DLC coating on a silicon substrate. X-ray photoelectron spectroscopy was performed using a FlexMod spectrometer (SPECS Surface Nano Analysis GmbH, Berlin, Germany) with an Mg Kα (1254 eV) excitation source and a PHOIBIS 150 hemispherical analyzer. The energy calibration involved referencing to adventitious carbon, the C 1s line located at 285.0 eV. High-resolution transmission electron microscopy (HRTEM) imaging, electron diffraction, energy dispersive X-ray (EDS), and electron energy-loss spectroscopy (EELS) were performed using a Talos-F200 microscope (FEI, Waltham, MA, USA) with an ‘XFEG’ Schottky field emission gun source at 200 keV. EELS scans were also performed with Talos using an electron current of 35 pA and a collection angle of 25 mrad; high-resolution scans were performed at 0.1 eV/channel. The moisture vapor transmission rate (MVTR) test was performed on Si-DLC coated and uncoated polymer materials to understand the functionality of Si-DLC coating for vapor transmission barrier applications. All of the samples were cut into 3.5-inch diameter circular swatches. Three uncoated polymer materials and three Si-DLC coated polymer materials were tested under standard atmosphere laboratory conditions of 21 ± 2 °C with 65 ± 5% relative humidity (RH). The moisture vapor diffusion rate through the Si-DLC coated and uncoated polymer materials was determined according to the Simple Dish Method, which is similar to ASTM E96-80. The samples are placed on water dishes with a diameter of 82 mm and a depth of 19 mm; a 9 mm air space was maintained between the polymer materials and the water surface. A vibration-free turntable containing eight dishes was rotated uniformly at a rate of 2 meters per minute; this approach ensured that all of the dishes were subjected to identical average ambient conditions during testing. The assembled specimen dishes were permitted to stabilize for 2 h prior to measurement of the initial weight. The assembled specimen dishes were weighed again after a 24-hour interval. The moisture vapor loss rate (MVTR) was calculated from these measurements in units of g/m^2^-24 h. For the water stability experiments, Si-DLC coatings on silicon substrates were immersed in a deionized (DI) water bath for four hours. For one set of the samples, the water temperature was kept at room temperature; for the other set of the samples, the water bath was maintained at 100 °C using a hot plate.

## 3. Results and Discussions

The PECVD process provides several advantages over other CVD processes. The plasma component in the PECVD process facilitates the decomposition of the gaseous precursors and reduces the substrate temperature that is required for the coating process [11,12]. Hence, the PECVD process is compatible with the growth of coatings on polymer and other heat-sensitive substrates. In addition, the PECVD process enables the deposition of coatings with controlled compositions and provides straightforward control over the reaction parameters [11,12,13]. PECVD allows for the conformal deposition and step coverage of substrates; a uniformly-shaped plasma may allow for the deposition of coatings over large areas [12,14]. Plasma pretreatment may be performed prior to deposition of the PECVD coating; plasma pretreatment increases the surface energy of the substrate by eliminating organic contaminants. The adhesion of the coating to the substrate may be enhanced by plasma pretreatment [15]. During the PECVD deposition process, the bombardment of the substrate with energetic particles increases the adhesion of coating through energetic particle implantation and enhanced surface mobility [16]. In addition, the PECVD process allows for precise control over the coating thickness [17]. As such, PECVD is an appropriate approach for the deposition of Si-DLC coatings over a large area on heat-sensitive polymer substrates. It should be noted that the PECVD process has several limitations. It requires complicated and expensive equipment. During the PECVD process, the buildup of toxic and explosive gaseous byproducts must be controlled. The PECVD process also suffers from the slower rate of growth than other methods such as pulsed laser annealing [18,19].

**Figure 2:**
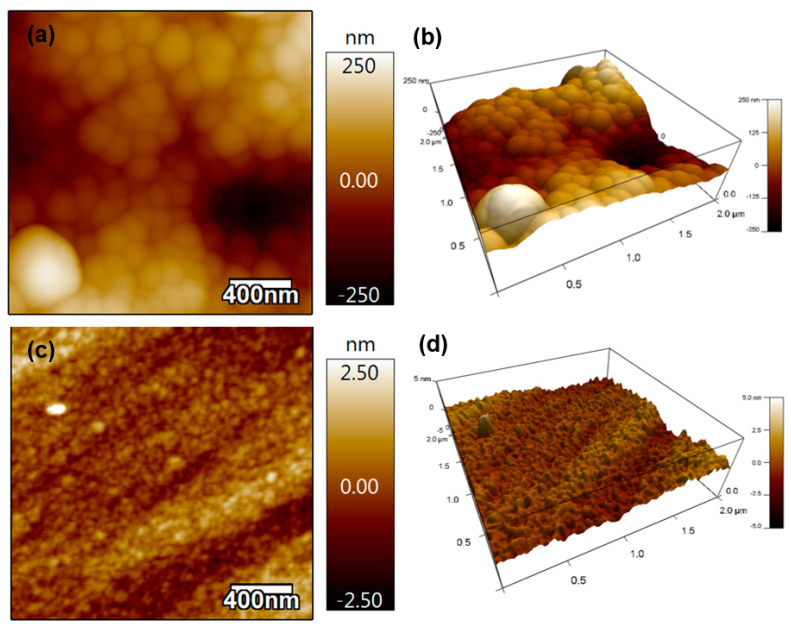
(**a**) The 2D AFM image of the Si-DLC coating on the polymer material, (**b**) the 3D AFM image of Si-DLC coating on the polymer material, (**c**) the 2D AFM image of Si-DLC coating on the Si substrate, and (**d**) the 3D AFM image of Si-DLC coating on the Si substrate.

The thickness of the Si-DLC coatings on Si substrates was measured at five random positions on the coating; the measured thickness value was 380 ± 19 nm. This thickness value corresponds to a deposition rate of ~6 nm/minute. Figure 2 contains the 2D and 3D AFM results from Si-DLC coatings on the polymer material (Figure 2a,b) and Si substrate (Figure 2c,d). No pinholes were noted on the coated surfaces. The root-mean-square (RMS) roughness values over a 2 µm × 2 µm surface area of the Si-DLC coating on the polymer material and the Si-DLC coating on the Si substrate were 86.5 ± 45 nm and 0.58 ± 0.3 nm, respectively. The RMS values of the uncoated polymer substrate and silicon substrate were 10.658 ± 8 nm and 0.7 +/− 0.2 nm, respectively. The low RMS roughness values of the Si-DLC coating on both substrates indicated the uniformity of the coating.

**Figure 3:**
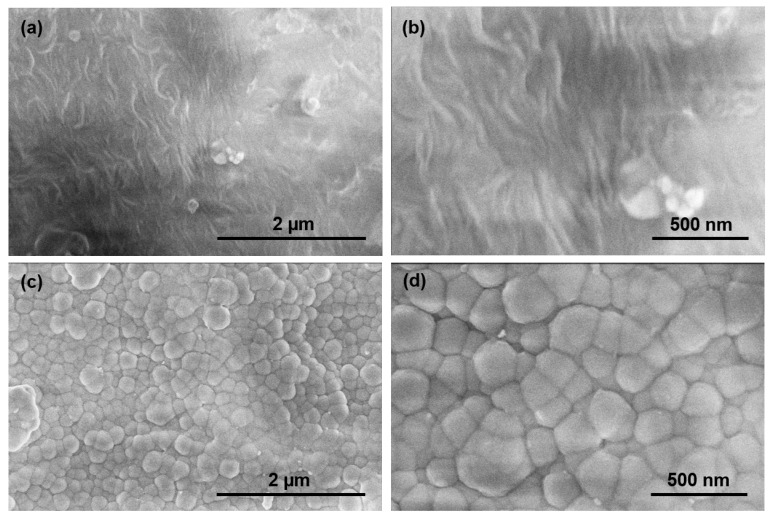
(**a**,**b**) FESEM images of the uncoated polymer material. (**c**,**d**) FESEM images of the Si-DLC coated polymer material.

Figure 3a,b contain FESEM images of the uncoated polymer material; Figure 3c,d contain FESEM images of the Si-DLC coated polymer material. The granular features exhibit an average size of 200 nm; the images also indicate the uniformity of the Si-DLC coating. No flaking of the coating or uncoated areas was observed over an area of ~15 µm^2^ in Figure 3c. These results are consistent with previous micro-scratch testing of the Si-DLC coating on fused silica, which revealed a high critical load for 2.961 ± 0.292 N and chipping as the only mode of failure [7]. The presence of Si in the Si-DLC coating may serve to enhance the adhesion of the Si-DLC coating to the substrate [20,21]; for example, the presence of Si may also enhance film adhesion through the reduction in stress in the Si-DLC coating [22].

**Figure 4:**
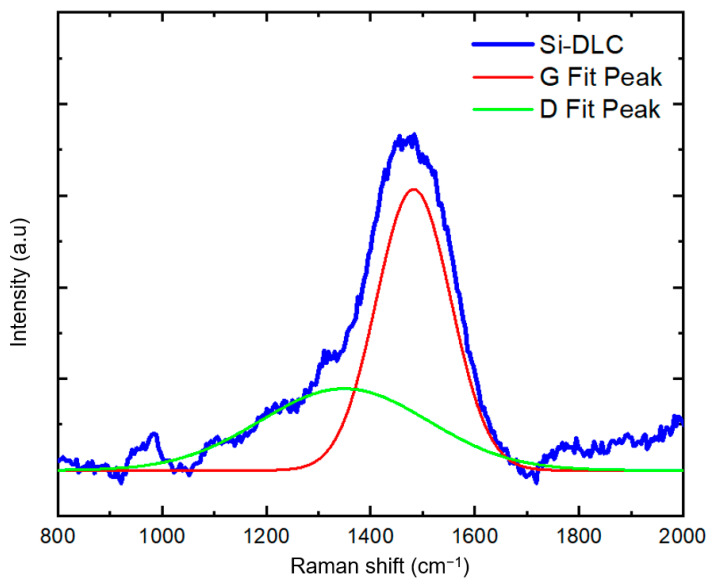
Raman spectrum obtained from the Si-DLC coating on a silicon substrate.

Raman spectroscopy is a powerful tool to understand carbon bonding in carbon-containing coating. Figure 4 contains the Raman spectrum obtained from the Si-DLC coating on the silicon substrate over the range of 800 to 2000 cm^−1^. Since distinguishing between the Raman spectral features associated with carbon bonding in the polymer material and the Si-DLC coating is difficult, we acquired the Raman spectrum from a Si-DLC coating on a silicon substrate. The peak around 1000 cm^−1^ is attributed to the silicon substate. In DLC coatings, the Raman spectrum takes the shape of a broad band between 1100 cm^−1^ and 1700 cm^−1^, which is comprised of two peaks at approximately 1350 cm^−1^ and 1580 cm^−1^. The D peak at ~1350 cm^−1^ is assigned to the A_1g_ symmetric breathing mode; the G peak at ~1580 cm^−1^ is assigned to the zone-center mode of E_2g_ symmetry [23,24,25]. The visible wavelength excitation source used in this study preferentially resonates with π states. Therefore, the sp^2^ bonds (π bonding) are detected 50 to 230 times more strongly than the sp^3^ bonds (σ bonding); the D peak appears as a shoulder peak [7,23,25]. In Figure 4, a Gaussian distribution was utilized for the deconvolution of the D and G peaks from the broad DLC band; the D and G fit-peaks are located at 1349 cm^−1^ and 1482 cm^−1^, respectively. In comparison with DLC coatings, Si-DLC structures show a downshift in the positions of the D and G peaks [26]. There are several reasons for the downshift of the D and G peaks with the incorporation of silicon in the DLC coating. In contrast with carbon atoms that form three-fold- and four-fold-coordinated bonds, silicon atoms form four-fold-coordinated bonding in DLC structures. Since silicon cannot form π-bonds when present in DLC structures, the sp^3^ to sp^2^ ratio in Si-DLC coatings increases, and the size of the graphite-like domains is reduced. The enhancement in sp^3^ content with the addition of silicon also reduces the internal residual stress in the DLC coatings. Consequently, silicon incorporation increases the adhesion of the Si-DLC coating to the substrate [15]. Therefore, the downshift in the position of D and G peaks in Si-DLC films is partially attributed to the reduction in the internal compressive stress that is associated with the presence of the silicon atoms. In other words, the vibration of the de-strained bonds occurs at lower frequencies. Moreover, the addition of silicon to the DLC coatings weakens the carbon–carbon bonds through the formation of silicon–carbon bonds, which culminates in a downshift in the frequency of D and G peaks and a reduction in the I_D_/I_G_ ratio [27].

**Figure 5:**
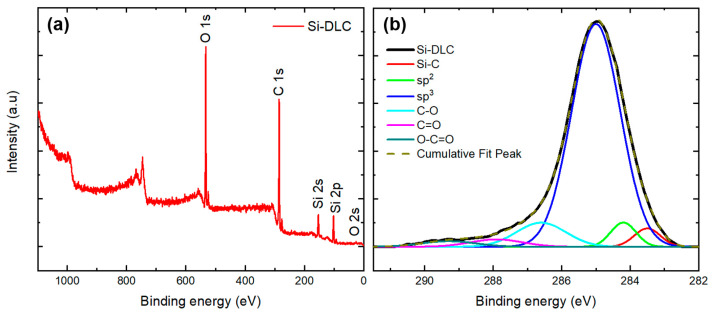
(**a**) XPS spectrum of the Si-DLC coating on a silicon substrate. (**b**) Deconvolution of the high-resolution C 1s band of the Si-DLC coating.

X-ray photoelectron spectroscopy (XPS) was used to obtain information on the elemental composition and the carbon bonding on the surface of the Si-DLC coating. Figure 5a shows the spectrum from the Si-DLC coating and Figure 5b shows the deconvolution of the high-resolution C 1s band. The Shirley model was used for the background determination [28]. The atomic percentages are presented in Table 1. The results indicate the absence of impurities on the surface and provide the elemental compositions for carbon, silicon, and oxygen. The deconvolution of the C 1s peak (Figure 5b) was used to obtain bonding information for the Si-DLC coating. The sp^2^-hybridized carbon bonds (284.2 eV), sp^3^-hybridized Si-C bonds (283.5 eV), sp^3^-hybridized carbon bonds (C-C at 285 eV), C-O bonds (286.6 eV), C=O bonds (287.9 eV), and O-C=O bonds (289.3 eV) were noted to be present on the surface of Si-DLC coatings; previous studies have also described the presence of these bonds in Si-DLC coating [29,30,31].

**Figure 6:**
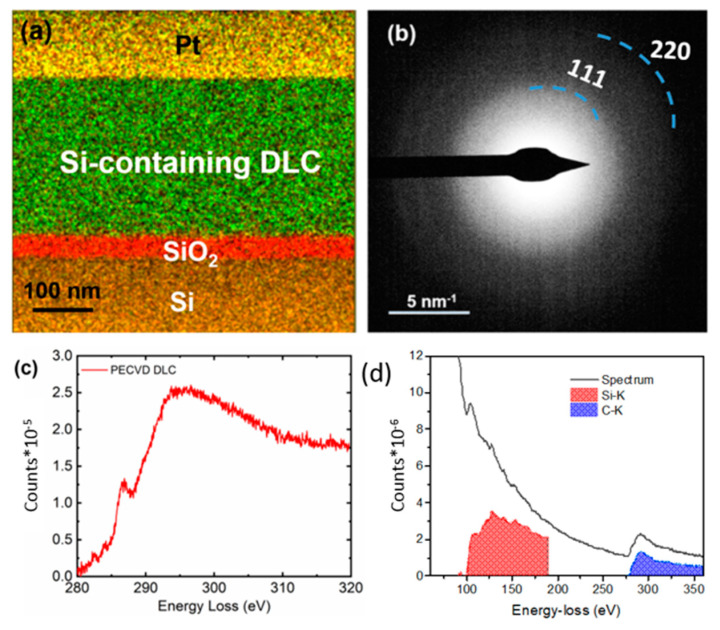
(**a**) Energy dispersive X-ray analysis of the Si-DLC coating with Si (in ochre), oxygen (in red), carbon (in green), and Pt (in yellow). (**b**) Selective area diffraction pattern from the Si-DLC coating, indicating the amorphous nature of the coating. (**c**) Electron energy loss spectrum from the Si-DLC coating. (**d**) Electron energy loss spectrum containing Si (red) and C (blue) edges.

A sample of the Si-DLC coating on the silicon substrate was prepared by focused ion beam (FIB) processing; EDS data was collected from the FIB-processed sample (Figure 6a). The elements were mapped in different colors; Si (in ochre), oxygen (in red), carbon (in green), and Pt (in yellow). The presence of Si was observed in the Si-DLC coating. The selective area diffraction (SAD) pattern from the Si-containing DLC coating is shown in Figure 6b. A small SAD aperture was used such that it only covered the Si-DLC coating; no stray signal from the Si/SiO_2_ interface or Pt was noted. The diffraction shows characteristic carbon (111) and (220) rings. The diffused rings confirm the amorphous nature of the Si-DLC coating.

Three major edges were observed in the EELS spectrum: C=C (π*) at 285 eV, C=C(σ*) at 292 eV, and C-C (σ*) at 298 eV (Figure 6c) [32]. Previous studies [33,34,35] have shown that the ratio of π*/σ* determines the sp^2^ content of the as-deposited DLC coating. Diamond exhibits a σ*peak (but no π*peak), whereas graphite shows the presence of both peaks. Gaussian fitting was performed on the C K-edge to estimate the sp^3^ content of the Si-DLC coating. To address the sample thickness-related plural scattering effects on the C K edge, the estimations were done after performing thickness corrections in the EELS spectrum using a zero-loss spectrum [36]. The Si-DLC coating was determined to contain an sp^3^ content of ~55% (Figure 6c). By performing further quantification of EELS spectra containing Si K (99 eV) and C K (284 eV) edges as shown in Figure 6d, we estimated ~18 ± 2% Si in the Si-DLC coating; this value is consistent with the results obtained by XPS. 

The vapor transmission performance of the Si-DLC coated polymer material was compared with that of the uncoated polymer material. MVTR is used to describe the performance of the material to protect against moisture; a higher MVTR value is associated with a greater passage of moisture vapor through the material. Table 2 summarizes the MVTR results for the Si-DLC coated polymer material and the uncoated polymer material. The average MVTR value for the three uncoated polymer materials was measured to be 10.10 (g/m^2^ 24 h), whereas the average MVTR value for the three Si-DLC coated polymer materials was 6.31 (g/ m^2^ 24 h). The decrease in the MVTR value for the Si-DLC coated polymer material of about 38% is associated with the moisture barrier performance of the Si-DLC coating.

**Figure 7:**
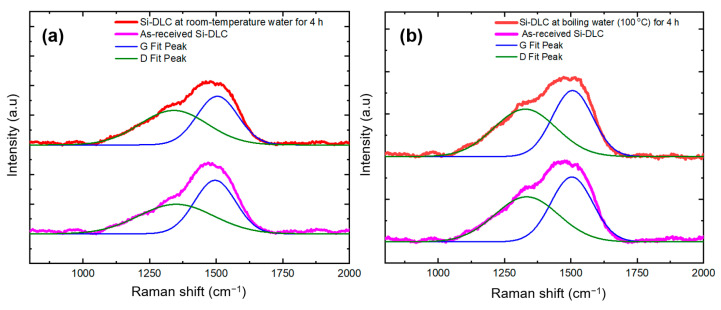
(**a**) The Raman spectra of the Si-DLC coating before and after soaking in DI water at room temperature for four hours, and (**b**) the Raman spectra of the Si-DLC coating before and after soaking in DI water at 100 °C for four hours.

To further investigate the capabilities of the Si-DLC coating related to protection against moisture, we carried out a water stability study. To perform the water stability study, the Si-DLC coatings on silicon substrates were immersed for four hours inside a deionized-filled water vessel that was kept at either room temperature or 100 °C. Raman spectroscopy was conducted on the samples before and after water immersion. Figure 7 shows the Raman spectra of the Si-DLC coating before and after soaking in DI water at room temperature for four hours as well as the Raman spectra of the Si-DLC coating before and after soaking in DI water at 100 °C for four h. As indicated in Figure 7a, the Si-DLC coating showed no change in spectral features after four hours of immersion in room temperature DI water. No significant alteration to the Si-DLC structure as indicated by abrupt changes to the D peak and G peak values was observed. The values of the D peak and G peak before water immersion at room temperature were 1349 cm^−1^ and 1495 cm^−1^, respectively; the values of the D peak and G peak after water immersion at room temperature were 1340 and 1504 cm^−1^, respectively. We also observed that the Si-DLC structure remained stable in contact with boiling water at a temperature of 100 °C for four hours (Figure 7b). Considering the resolution limitation of the Raman instrument (5 cm^−1^), the structure showed similar values for the D peak and G peak before and after immersion in water at 100 °C. The values of the D peak and G peak before water immersion at 100 °C were 1333 cm^−1^ and 1504 cm^−1^, respectively; the values of the D peak and G peak after water immersion at 100 °C were 1327 and 1506 cm^−1^, respectively. We also performed a peel-off adhesion test using scotch tape on the Si-DLC coating before and after water immersion. The results indicated the high-quality adhesion of the Si-DLC coating to the silicon substrates after immersion in 100 °C water for 4 h.

## 4. Conclusions

We deposited Si-DLC coatings on commercial polymer materials in a cylindrical shape with large dimensions, specifically a diameter of 20.3 cm and a height of 17.8 cm. We have shown that PECVD method is a suitable approach for depositing large area Si-DLC coatings. The MVTR studies indicated that the Si-DLC film can serve as a moisture barrier on polymer materials. The uniformity of the Si-DLC coating with no flaking, as indicated by AFM and FESEM studies, and the absence of contaminants, as indicated by the XPS study, indicate that this material holds promise for commercial moisture-resistant packaging applications. The water stability results show the stability of the structural stability of the Si-DLC coatings in contact with moisture up to 100 °C.

## Figures and Tables

**Table 1:** The XPS curve fitting results from C 1s, Si 2p, and O 1s peaks corresponding with Figure 5a.

Coated Layer	Parameter	Peaks in XPS Spectrum		
		C 1s	Si 2p	O 1s
Si-DLC	Position (eV)	285.3	101.8	532.8
	FWHM (eV)	2.14	2.87	2.01
	Atomic %	59.1	16	24.9

**Table 2:** Moisture vapor transmission results for the uncoated polymer material and the Si-DLC coated polymer material.

Sample	Weight 1 (grams)	Weight 2 (grams)	W1-W2 (grams)	WVT (g/h m^2^)	MVTR (g/ m^2^ 24 h)
Uncoated polymer (1)	139.90	139.84	0.06	0.47	11.36
Uncoated polymer (2)	138.39	138.33	0.06	0.47	11.36
Uncoated polymer (3)	140.63	140.59	0.04	0.32	7.57
**Avg.**			**0.05**	**0.42**	**10.10**
Si-DLC coated polymer (1)	138.27	138.23	0.04	0.32	7.57
Si-DLC coated polymer (2)	137.58	137.55	0.03	0.24	5.68
Si-DLC coated polymer (3)	137.32	137.29	0.03	0.24	5.68
**Avg.**			**0.03**	**0.26**	**6.31**

## Data Availability

The datasets generated during and/or analyzed during the current study are available from the corresponding author on reasonable request.
